# The Best Choice of Treatment for Acute Colonic Diverticulitis with Purulent Peritonitis Is Uncertain

**DOI:** 10.1155/2014/380607

**Published:** 2014-06-03

**Authors:** Line Hupfeld, Jakob Burcharth, Hans-Christian Pommergaard, Jacob Rosenberg

**Affiliations:** Center for Perioperative Optimization, Department of Surgery, Herlev Hospital, University of Copenhagen, Herlev Ringvej 75, 2730 Herlev, Denmark

## Abstract

Severe stages of acute, colonic diverticulitis can progress into intestinal perforations with peritonitis. In such cases, urgent treatment is needed, and Hartmann's procedure is the standard treatment for cases with fecal peritonitis. Peritoneal lavage may be an alternative to resection for acute diverticulitis with purulent peritonitis, but ongoing randomized trials are awaited to clarify this.

## 1. Introduction

Diverticular disease is a common gastrointestinal condition. Diverticula inflammation develops in 10–25% with diverticular disease causing acute diverticulitis [1] and it is the fifth most expensive gastrointestinal disease in America [2]. Acute diverticulitis occurs in an uncomplicated stage with local, colonic phlegmonous inflammation and a complicated form graded by the Hinchey classification [3].

Acute diverticulitis is treated according to severity. Treatment recommendations include conservative approaches with observation and dietary modifications along with antibiotic treatment, abscess drainage, and surgery. A differentiated treatment dependent on disease stage is agreed upon in current national and international guidelines [4, 5]. However, the specific choice of treatment varies and no consensus seems to be present at this point, especially when managing the most severe cases of acute diverticulitis with bowel perforation, where urgent treatment is required to prevent sepsis and possible death.

We aimed to evaluate current treatments for perforated acute diverticulitis based on the available literature in order to determine the most effective and protective approach.

## 2. Classification

**Figure 1:**
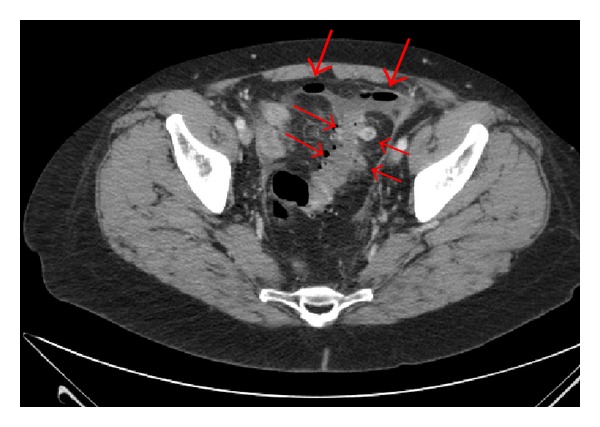
CT scan of perforated diverticulitis with diverticula (thin arrows) and free abdominal air (thick arrows).

Acute, complicated diverticulitis is divided into four stages according to the Hinchey classification, based upon preoperative findings of abscesses and intestinal perforation (Table 1) [3]. In the most severe cases, abscess perforation leads to purulent peritonitis (Hinchey 3) and diverticula rupture to faecal peritonitis (Hinchey 4) (Figure 1).

## 3. Treatment of Acute Colonic Diverticulitis with Perforation, Hinchey Stage 3 + 4

Perforated colonic diverticulitis is treated by surgical intervention. The standard treatment is the Hartmann procedure (resection of the diverticula affected colonic segment, closure of the rectal stump, and formation of an end colostomy [6]). In the elective setting, a laparoscopic approach is preferred since it provides less pain and a faster recovery [5]. In acute settings, however, the choice of surgical approach varies greatly around the world, where the availability of necessary expertise for acute laparoscopic procedures can be a limiting factor, especially during night hours.

### 3.1. The Different Surgical Procedures

Over the years, alternative surgical methods have been explored. A review summarizing former surgical methods for acute diverticulitis described the use of a three-stage procedure with firstly diverting colostomy and suture of the intestinal perforation, secondary colonic resection, and lastly stoma reversal [7]. Also, colonic resections with primary anastomosis with or without a proximal diverting ileostomy and on-table colonic lavage [8] have been evaluated. Results from clinical studies were not convincing and most methods are no longer recommended; yet the role of primary anastomosis in diverting ileostomy, as an alternative to Hartmann's procedure, remains unsettled [5].

### 3.2. Experiences with Peritoneal Lavage

Within the last decades, a more conservative approach of peritoneal lavage has been investigated as an alternative to colonic resection. In this procedure, pus is aspirated typically by laparoscopic access followed by abdominal lavage with heated saline and drainage for some days after the procedure [9]. So far, no randomized controlled clinical trial has been published comparing peritoneal lavage with colonic resection in the treatment of perforated diverticulitis.

One of the largest studies on peritoneal lavage was published in 2007 [9]. This prospective multicenter study included 100 patients with radiological verified complicated diverticulitis (Hinchey 2–4). Patients were preoperatively treated with iv fluid and antibiotics before undergoing laparoscopic peritoneal lavage and drainage. Findings of fecal peritonitis led to Hartmann's procedure. Antibiotic treatment continued for minimum 72 hours postoperatively and oral intake of fluids was restricted on the first postoperative day. Laparoscopic peritoneal lavage was performed in 92 patients (Hinchey 2 + 3) and eight had a Hartmann procedure (Hinchey 4). Of the 92 patients treated with lavage, three patients died (two of multiple organ failure, one of pulmonary embolism), whereas two patients were immunosuppressed following a renal transplant. The clinical resolution was absent in two patients, who proceeded to colonic resection (one patient) and radiologic abscess drainage (one patient). At the following colonoscopy, diverticular disease and inflammatory resolution were confirmed in all patients experiencing resolution after peritoneal lavage. Within a median follow-up period of 36 months, two patients were readmitted with acute diverticulitis, both managed with antibiotic treatment. Conclusively, lavage was recommended as a reasonable alternative to Hartmann's procedure for perforated diverticulitis, Hinchey 3, in order to avoid major surgery and stoma formation.

A prospective database study followed 88 patients with perforated diverticulitis (Hinchey 2–4) after receiving acute treatment of laparoscopic colonic resection or laparoscopic peritoneal lavage with drainage [10]. Patients undergoing diagnostic laparoscopies were excluded due to a criterion on preoperative CT verification of the perforation. A total of 47 patients received peritoneal lavage and 41 underwent a Hartmann procedure. The distribution of demographics, disease severity (Hinchey 2–4), and comorbidities was comparable between the groups. Peritoneal lavage was significantly faster, with less blood loss and intraoperative complications compared to Hartmann's procedure. One lavage procedure was converted to a Hartmann procedure. Furthermore, postoperative complications differed significantly between 4.3% and 12.5% after peritoneal lavage and Hartmann's procedure, respectively. One patient died after a Hartmann procedure. Patients were hospitalized significantly longer after Hartmann's procedure than after laparoscopic lavage. During follow-up, 21 of 47 lavage patients had a secondary sigmoidectomy performed for source control. The remaining 26 did not receive further treatment. Stoma closure secondary to the Hartmann procedure was performed in 72% of patients. Conclusively, peritoneal lavage and Hartmann's procedure were both considered suitable for acute inflammatory control. Despite the fact that the pathological source was not removed, peritoneal lavage had significantly better short- and long-term outcomes compared with laparotomy. However, almost half of these patients had subsequent surgical resection.

A retrospective study of 38 patients with perforated, purulent diverticulitis treated with peritoneal lavage was published as a preliminary evaluation before the start of the ongoing randomized Ladies trial [11]. Patients between 18 and 85 of age with radiologically verified perforated diverticulitis and eligible for surgical treatment were included. Peritoneal lavage was successful in 31 of 38 patients with inflammatory resolution. Of the 31 patients managed successfully with lavage, three patients underwent subsequent sigmoid resection for recurrent attacks after six, nine, and twelve months, respectively, and one patient died due to a coexisting inoperable lung carcinoma. Patients unresponsive to peritoneal lavage showed a tendency towards greater comorbidity, higher preoperative CRP levels, and higher Mannheim Peritonitis Index scores. The authors concluded that peritoneal lavage could be a feasible alternative for a selected group of patients with Hinchey 3 diverticulitis and stressed the need for further research on the selection of eligible patients before general implementation. Furthermore, they concluded that the use of lavage in Hinchey 4 was unsafe.

## 4. Ongoing Studies and Preliminary Results 

Currently, four ongoing randomized clinical trials investigate the potential of peritoneal lavage compared with Hartmann's procedure for acute perforated diverticulitis [12–15]. As for now, no preliminary results from these five studies have been published.

In 2013, an American group presented preliminary results on the use of laparoscopic peritoneal lavage for complicated diverticulitis [16]. Ten patients underwent a diagnostic laparoscopy after insufficient response to antibiotic treatment. In one patient, laparoscopic findings of fecal peritonitis led to resection with primary anastomosis and diverting ileostomy. Another procedure was converted to Hartmann's resection after findings of widespread intra-abdominal purulence. The remaining eight patients had a laparoscopic peritoneal lavage performed until drainage water was clear. Intravenous antibiotics and fluids were administered postoperatively with conversion to oral antibiotics and solid diet based on the clinical status. Four patients were subsequently readmitted with recurrent episodes. Two patients underwent secondary sigmoid resection, whereas two were diagnosed with adenocarcinoma.

Complete results from the ongoing trials are awaited and may determine whether peritoneal lavage can be a future bowel-preserving treatment modality for purulent, perforated acute diverticulitis.

## 5. Current Recommendations

As for now, no level 1 evidence has been published on the use of peritoneal lavage. Current evidence does, however, advocate for peritoneal lavage as a safe alternative to Hartmann's procedure for purulent, perforated diverticulitis (Hinchey 3), at least in selected patients [9, 17–19]. Further knowledge is needed on the long-term morbidity and mortality as well as health economic parameters before general implementation can be considered [9, 19–21]. Peritoneal lavage is not recommended in cases of pelvic abscesses or fecal peritonitis due to a high risk of treatment failure [17–19].

Official recommendations on the use of peritoneal lavage differ. Treatment guidelines for sigmoid diverticulitis by the American Society of Colon and Rectal Surgeons briefly mention peritoneal lavage as a possible supplement during surgical resections [5], whereas the Danish National Guidelines recommend peritoneal lavage as the primary treatment for complicated, purulent diverticulitis along with antibiotic treatment and abscess drainage [4]. Worldwide, Hartmann's procedure remains the treatment of choice in treating fecal perforated diverticulitis.

## 6. Potential Treatment Algorithm for Acute Diverticulitis

**Figure 2:**
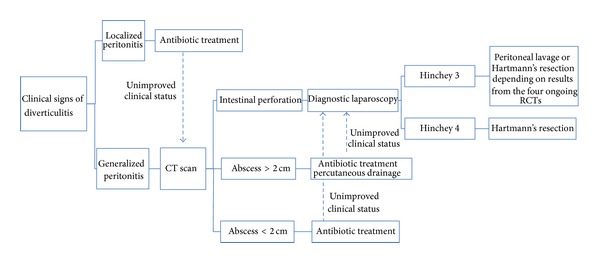
Potential treatment algorithm for acute diverticulitis in hospitalized patients.

Along with the preliminary results, an algorithm for the treatment of patients hospitalized with diverticulitis was suggested in a recent publication [16]. Within this algorithm, peritoneal lavage was incorporated as a standard approach for purulent diverticulitis (Hinchey 3). We have adapted the algorithm to the available evidence and current traditions (Figure 2): patients with suspected acute diverticulitis are operated on according to clinical signs of local or generalized peritonitis and hemodynamic stability. Patients being hemodynamically unstable or with signs of shock, where it is not possible to stabilize them with standard fluid regimens, must urgently be taken to surgery. Hemodynamically stable patients with clinical signs of generalized peritonitis could undergo CT imaging. Findings of diverticulitis Hinchey stage 3 lead to peritoneal lavage if supported by the five ongoing RCTs, and Hartmann's resection must be offered in cases of fecal peritonitis (Hinchey 4). Patients with clinical signs of generalized peritonitis and CT findings of abscesses larger than 2 cm should undergo percutaneous abscess drainage after receiving broad-spectrum antibiotics. In cases of unimproved clinical status, a diagnostic laparoscopy is performed. Antibiotics can be received when CT findings of abscesses smaller than 2 cm or abscesses inaccessible for drainage can receive antibiotics exist. If the clinical status continues without improvement, a diagnostic laparoscopy can be considered. Patients with suspected diverticulitis and a local peritoneal reaction should receive antibiotic treatment. If the clinical status does not improve, CT imaging must be performed.

## 7. Conclusion

Perforated acute diverticulitis is treated by surgical intervention. Worldwide, Hartmann's procedure remains the gold standard and the primary choice for acute diverticulitis with fecal peritonitis. Peritoneal lavage is a more conservative and bowel-preserving approach compared to resection in purulent diverticulitis. Peritoneal lavage has currently been added to certain official treatment guidelines. The lack of level 1 evidence does, however, keep peritoneal lavage from being implemented as a routine treatment. Current ongoing randomized trials on surgical treatments for perforated diverticulitis are awaited to determine if peritoneal lavage can be recommended as a routine approach. When data from these are available, guidelines may be adjusted. In the meantime, treatment must be decided on an individual basis when treating acute perforated, colonic diverticulitis.

## Figures and Tables

**Table 1:** Hinchey classification: grading of acute, complicated diverticulitis [3].

Hinchey stage	Complications to acute diverticulitis
1	Localized abscess (para-/mesocolic)
2	Pelvic abscess
3	Purulent peritonitis
4	Feculent peritonitis
